# Morphological, Genetic, and Microbiological Characterization of *Tuber magnatum* Picco Populations from “Alto Molise”, Central-Southern Italy

**DOI:** 10.3390/microorganisms13102340

**Published:** 2025-10-11

**Authors:** Antonio Bucci, Pamela Monaco, Claudio Caprari, Danilo Di Pilla, Antonietta Mello, Gabriella Sferra, Gino Naclerio

**Affiliations:** 1 Department of Biosciences and Territory, University of Molise, C.da Fonte Lappone, 86090 Pesche, Italy; antonio.bucci@unimol.it (A.B.); pamela.monaco@unimol.it (P.M.); claudio.caprari@unimol.it (C.C.); danilo.dipilla@unimol.it (D.D.P.); naclerio@unimol.it (G.N.); 2 Turin Unit, Institute for Sustainable Plant Protection (IPSP), National Research Council, Viale P.A. Mattioli 25, 10125 Turin, Italy

**Keywords:** ectomycorrhizal fungi, truffle, *Tuber magnatum* Picco, ascocarp-associated microbial communities, Molise region

## Abstract

The Molise region in Central-Southern Italy is a major contributor to national truffle production, particularly of the highly prized *Tuber magnatum* Picco, accounting for approximately 40% of the country’s total output and hosting the highest density of truffle harvesters. Despite this, research on the Italian white truffle populations from this area remains limited. Therefore, the primary objective of the present study was to address this knowledge gap by characterizing four *T. magnatum* Picco populations collected from the municipalities of Agnone, Carovilli, Castel del Giudice, and Pietrabbondante, located in “Alto Molise”, through morphological, genetic, and microbiological investigations. The statistical analyses revealed significant differences in peridium thickness and ascocarp-associated microbiota even though pairwise comparisons did not identify statistically significant differences between specific population pairs. No significant variation was observed in ascocarp weight and maturation degree. Furthermore, the presence of a unique haplotype at the single-locus marker SCAR A21-inf was confirmed in a subset of the analyzed fruiting bodies. Collectively, these findings expand current biological knowledge of the Molise white truffle and provide a foundation for future research aimed at identifying specific provenance markers to discriminate truffle populations at both regional and local scales.

## 1. Introduction

Fungi of the genus *Tuber* (commonly referred to as true truffles) are Ascomycetes within the order *Pezizales*, a diverse group of ectomycorrhizal fungi that form symbiotic associations with the roots of various vascular plants, including both angiosperms and gymnosperms [1,2,3,4,5].

The genus *Tuber* comprises an estimated 180–220 species [5,6], though only a small subset produces ascomata with notable gastronomic and economic value. Among the most commercially important species are *Tuber magnatum* Picco (the Italian white truffle), *Tuber melanosporum* Vittad. (the Périgord black truffle), *Tuber aestivum* Vittad. (summer black truffle), and *Tuber borchii* Vittad. (bianchetto truffle). Market prices for these species range from 30 to 6000 euros per kilogram, influenced by several factors such as species identity, ascomata size, harvest season, maturity, and availability [5]. *Tuber magnatum*, in particular, is renowned for its intense aroma and distinctive flavor, making it the most highly valued among truffle species. Consequently, it commands the highest retail prices in Italy and is considered one of the most expensive food products worldwide [5,7].

Elevated truffle costs have prompted various forms of adulteration [8], including the substitution of the premium species *T. magnatum* with lower-value ones such as *T. borchii* [9,10]. Moreover, the commercial price of *T. magnatum* can exhibit significant variation depending on its geographical provenance, even within local or regional scales [11,12]. This variability could enable the marketing of fruiting bodies harvested from areas considered less prestigious at prices comparable to those from more esteemed regions [13]. Compounding these issues, the current lack of rigorous regulatory frameworks governing the production, labeling, and marketing of truffle-derived products permits the use of the terms “truffle” or “truffled,” as well as the depiction of premium *Tuber* species on packaging, regardless of the actual species content or the inclusion of flavoring agents [10]. Supporting this concern, a recent investigation in Spain demonstrated that only 20% of truffle-based products were accurately labeled [10]. Such regulatory shortcomings contribute to consumer confusion, depreciate the value of this highly prized commodity, and negatively impact the interests of authentic producers [10]. Accordingly, the identification of specific markers indicative of *T. magnatum* geographic origin, coupled with a comprehensive characterization of its main features, could facilitate the recognition of region-specific traits. Such efforts would not only support traceability and authentication but also contribute to the conservation and valorization of this highly valuable biological resource, which plays a critical role in sustaining certain local economies.

Although numerous studies have predominantly focused on the prized *T. magnatum* Picco, the Molise white truffle remains comparatively understudied [4,14,15,16]. Nonetheless, the Molise region is among the richest Italian areas for truffle production [17], particularly for the valuable *T. magnatum*, contributing approximately 40% to the national truffle yield. Furthermore, the proportion of truffle harvesters operating in the region is, relative to other regions, the highest in Italy [4].

To expand current knowledge on the Molise white truffles, the present study undertook an integrated morphological, genetic, and microbiological characterization of four *T. magnatum* populations collected from distinct municipalities within the “Alto Molise” area.

## 2. Materials and Methods

### 2.1. Study Areas and Sample Collection

**Figure 1:**
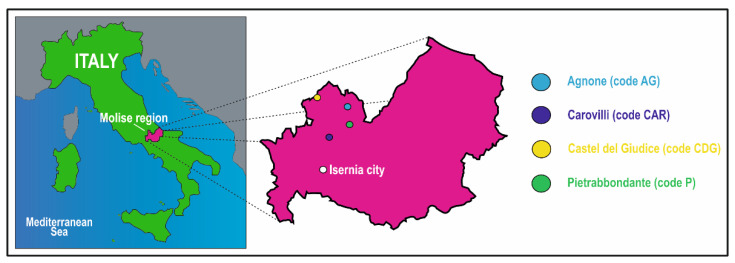
Location of the study areas. The colored circles denote the municipalities of Agnone (code AG), Carovilli (code CAR), Castel del Giudice (code CDG), and Pietrabbondante (code P) in the “Alto Molise” area, Molise region, Central-Southern Italy.

A total of twenty intact *T. magnatum* fruiting bodies were collected from four locations within the municipalities of Agnone (code AG), Carovilli (code CAR), Castel del Giudice (code CDG), and Pietrabbondante (code P) in the Molise region (Figure 1), between the end of November and the beginning of December 2024. The natural truffle grounds were located within mixed coppice woodlands, characterized by a vegetation composition of hazel trees (*Corylus avellana* L.), Turkey oaks (*Quercus cerris* L.), beeches (*Fagus sylvatica* L.), and cornels (*Cornus sanguinea* L.). Specifically, five ascocarps (numbered 1 to 5) were collected from each site, excavated with the assistance of trained truffle dogs and experienced personnel. The specimens were then individually placed in sterile polypropylene containers and transported to the laboratory under refrigerated conditions. *Tuber magnatum* ascomata were carefully brushed with a sterile soft brush and rinsed with sterile distilled water prior to morphological and molecular analyses.

Morphological analyses and PCR-based techniques were conducted to confirm species identification, assess the maturation stages of fruiting bodies, and quantify peridium thickness. For genetic variability assessment, the Sequence-Characterized Amplified Region (SCAR) A21-inf was analyzed to detect single nucleotide polymorphisms (SNPs). This single locus marker has previously shown to be polymorphic among *T. magnatum* ascocarps and enabled the identification of three distinct haplotypes. Finally, both the bacterial and fungal communities associated with Molise ascomata were characterized through Next-Generation Sequencing of 16S rRNA gene and ITS2 region amplicons. Additionally, network-based approaches, which have the potential to identify peculiarities of complicated communities associated with truffles [17,18], have been applied to reveal these traits across the examined truffle populations of Molise. Dried samples of each specimen were deposited in the herbarium at the University of Molise, Italy.

### 2.2. Ascocarp Morphological Characterization

**Figure 2:**
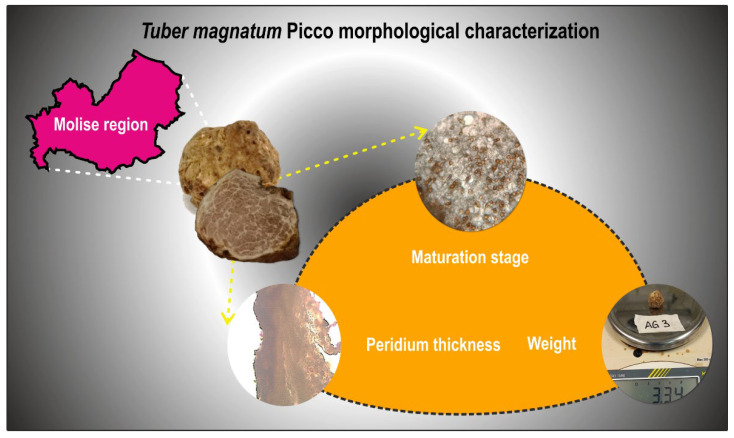
Schematic representation of the morphological characterization performed on the twenty ascomata collected from natural truffle grounds in the Molise region, aimed at determining their weight, maturity degree, and peridium thickness.

After removing any soil residue, each ascocarp was weighed using a precision scale (Figure 2). Then, fruiting bodies were halved: one half was used for peridium thickness and maturity assessment, whereas the other one was used for molecular analyses.

To assess the degree of maturation, five thin slices of fresh tissue taken from different portions of each ascoma were examined under a light microscope (ZEISS PrimoStar, Carl Zeiss Microscopy, Oberkochen, Germany) (Figure 2). Truffles were classified into three maturation stages based on the percentage of asci containing mature spores [19]: immature (0–5% asci with mature spores, Stage I), intermediate (6–60% asci with mature spores, Stage II), and mature (>60% asci with mature spores, Stage III). Spore maturation was determined morphologically, with mature spores appearing yellow-reddish brown and exhibiting reticulate ornamentation. The species identity of the truffles was confirmed as *T. magnatum* through both morphological characterization and PCR-based techniques [4,19].

To determine the peridium thickness, fruiting bodies were sliced lengthwise with a steel blade to obtain thin sections. For each truffle, five sections were sampled from different points of the ascoma, with each section also including a small portion of gleba to aid subsequent microscopic measurement of peridium thickness (Figure 2). The sections were placed on a microscope slide, wet with distilled water, and covered with coverslip. Peridium thickness was measured using a light microscope (ZEISS PrimoStar, 10× objective), equipped with a ruler, with measurements taken at 25 different points per fruiting body (five points per section). The average peridium thickness was calculated as the arithmetic mean of these 25 values [20].

To compare the four populations with respect to fruiting body weight, maturity stage, and peridium thickness, a Kruskal–Wallis test was applied to assess statistically significant differences among groups with the software package PAST version 5.2.1 [21]. Where significant variations were detected, post hoc pairwise comparisons were conducted using Dunn’s test with Bonferroni correction.

### 2.3. Molecular Analyses

#### 2.3.1. DNA Extraction from Fruiting Bodies

DNA was extracted from one half of the twenty white truffles to confirm their taxonomic identification, evaluate genetic variability, and analyze the composition of their associated bacterial and fungal communities. After removing the outermost portion of the gleba with a sterile steel blade, the inner portion was cut into small pieces [22,23]. These pieces were then shredded, and total genomic DNA was subsequently extracted using the DNeasy^®^ Plant Pro Kit (Qiagen, Hilden, Germany), in accordance with the manufacturer’s protocol.

#### 2.3.2. Assessment of *T. magnatum* Genetic Variability

The ITS regions of *T. magnatum* fruiting bodies were amplified using the specific primers P7 (5′-TCCTACCAGCAGTCTGAGAAAGGGC-3′) and M3 (5′-TGAGGTCTACCCAGTTGGGCAGTGG-3′), according to the protocol described by Mello et al. [24]. PCR products were visualized by agarose gel electrophoresis to molecularly confirm the sample identity as *T. magnatum* species.

The single locus marker SCAR A21-inf was amplified with the primer pair CL1 (5′-CTTGAGCAAACTCCAATAGAG-3′) and CL2 (5′-GACACGATCCAAGTCGAGAG-3′), following the PCR conditions reported by Mello et al. [22]. Then, the PCR products were purified with the “Wizard^®^ SV Gel and PCR Clean-Up System” kit (Promega, WI, USA) and subsequently sequenced at BMR Genomics srl (Padova, Italy). The resulting SCAR A21-inf sequences were aligned with CLUSTAL W (https://www.genome.jp/tools-bin/clustalw, accessed on 15 May 2025) and their chromatograms visually verified for SNPs detection using SEQUENCHER 5.4.6 (Gene Codes). Subsequently, the sequences were deposited in the GenBank database (accession numbers from PV754620 to PV754639). Fisher’s exact test was conducted to evaluate the association between provenance and haplotype with the software package PAST version 5.2.1 [21].

#### 2.3.3. Profiling of Microbial Communities Associated with Truffles Using 16S rRNA Gene and ITS2 Region Amplicon Sequencing and Bioinformatics

Sequencing analyses were performed at BMR Genomics srl (Padova, Italy). To investigate the prokaryotic communities associated with *T. magnatum* ascomata, starting from the total genomic DNA extracted from the gleba, the V3–V4 regions of the 16S rRNA gene were amplified using the primers Pro341F (5′-CCTACGGGNBGCASCAG-3′) and Pro805R (5′-GACTACNVGGGTATCTAATCC-3′), modified with universal tails [25].

The modified primers ITS3_KYO2 (5′-GATGAAGAACGYAGYRAA-3′) [26] and ITS4r (5′-TCCTCCGCTTATTGATATGC-3′) [27], instead, were used to amplify the ITS2 regions, in order to analyze the composition of fungal communities within truffle fruiting bodies.

A standard protocol routinely used by BMR Genomics Company (Padova, Italy) was employed for Next-Generation Sequencing. Briefly, PCR products were purified with Thermolabile Exonuclease I (New England Biolabs, Ipswich, MA, USA), diluted 1:2, and amplified with Nextera XT Index on a second PCR step. Amplicons were normalized with SequalPrep (Thermo Fisher, Waltham, MA, USA) and multiplexed. The pool was purified with Agencourt XP 1X magnetic beads. Lastly, the library was run on the Illumina MiSeq and sequenced with V3 chemistry—300PE strategy [28].

Bioinformatics analysis was performed using QIIME2 tools version 2024.10 [29,30]. The reads were cleaned of primers using the Cutadapt software (v. 2023.7) and then processed with the denoised-paired plugin of the DADA2 software [31]. Sequences were trimmed at the 3′ end, filtered by quality and length, dereplicated, and merged to obtain unique sequences. Lastly, chimeras were eliminated. The Amplicon Sequence Variants (ASVs) were filtered by length (thus eliminating potential “contaminant sequences”) and by frequency (0.001%) to remove poorly represented sequences and balance the ASV number across the analyzed samples. A rarefaction analysis was performed to determine the minimum number of reads required for sample normalization. All reads were classified to the lowest possible taxonomic rank using a reference dataset from the SILVA database (version 138.2) for bacteria and from the Unite database (version 9.0.99) for fungi.

The 16S rDNA and ITS2 sequences generated in the present study were deposited in the NCBI Sequence Read Archive (SRA) under the accession number PRJNA1272064.

Alpha-diversity was calculated with the Shannon index, and the Kruskal–Wallis test was applied to assess the significance of comparisons between truffle populations. Beta-diversity analyses were performed using the Bray–Curtis metric, while the statistical comparisons among and between experimental groups were conducted using the PERMANOVA pseudo-F statistic. Differential abundance at the genus level was assessed using the Analysis of Compositions of Microbiomes with Bias Correction (ANCOM-BC) method.

Microbial communities of each individual truffle population were modeled into co-occurrence networks based on sequence data. Significant co-occurrences at species level were identified by Pearson’s correlation, calculated with rcorr function from Hmisc package (version 5.2.2) [32] in R [33]. Only strong and statistically significant correlations were retained (r > 0.7, r < −0.7; *p*-value < 0.05). Species involved in these strong associations were considered due to their pronounced statistical connectivity and the related network analyses and visualizations were performed by igraph package [34] in R (version 4.4.1) and Cytoscape (version 3.10.1), respectively [35].

For each network, corresponding random networks—with the same number of nodes and edges—were generated using igraph package (100 iterations) and analyzed following the same procedure described above. The properties calculated on random networks were then averaged and showed together with their standard deviations. The betweenness centrality was considered as a parameter for evaluating the among-module connectivity (Pi) of taxa and identifying them as connectors if Pi > 0.62. Also the closeness centrality of each taxon was evaluated as within-module connectivity (Zi) identifying a taxon as a module hub if Zi > 2.5 [36]. Taxa that are highly connected with others both within and among modules are referred to network hubs [37].

## 3. Results

### 3.1. Identification and Morphological Features of T. magnatum Picco Ascocarps

The twenty ascocarps collected from the municipalities of Agnone (AG), Carovilli (CAR), Castel del Giudice (CDG), and Pietrabbondante (P) in the Molise region were identified as *T. magnatum* Picco based on their morphological features. Specifically, truffle coloration varied from pale yellowish-brown to yellow ochre, olivaceous, or greenish-gray, with some specimens displaying black or brownish surface spots. The peridium was smooth and suede-like, while the gleba—whitish to yellowish in immature ascocarps and hazel to brown in mature ones—showed numerous fine and clear veins [38]. This morphological identification was corroborated by molecular analysis: indeed, the amplification of the ITS regions using *T. magnatum*-specific primers [24] confirmed that all the examined fruiting bodies belonged to this species.

As shown in Table 1, the weight of the ascocarps ranged from 2.75 g (sample P2 from Pietrabbondante) to 7.83 g (sample CDG3 from Castel del Giudice). The mean weights for the white truffle populations from Agnone, Carovilli, Castel del Giudice, and Pietrabbondante were 4.60 g, 4.82 g, 5.97 g, and 5.13 g, respectively. However, the Kruskal–Wallis test revealed no significant differences in terms of weight among the four populations (*p* > 0.05).

As regards the maturation degree of the ascocarps, all five samples collected from the municipality of Pietrabbondante were classified as mature (Table 1). For Castel del Giudice, three ascomata exhibited an intermediate ripeness stage, while the remaining two were mature. The populations of *T. magnatum* Picco from Agnone and Carovilli included ascocarps representing all three maturation stages. Also regarding the maturation degree of truffles, the Kruskal–Wallis test indicated no significant differences among the investigated populations (*p* > 0.05).

Peridium thickness ranged from 182.80 μm in sample P3 to 394.80 μm in sample AG5 (Table 1). The mean peridium thickness for the populations from Agnone, Carovilli, Castel del Giudice, and Pietrabbondante were 319.02 μm, 261.58 μm, 234.22 μm, and 229.33 μm, respectively. The Kruskal–Wallis test revealed a significant difference among the four populations analyzed (*p* < 0.05). However, the subsequent pairwise comparisons using Dunn’s test with Bonferroni correction did not show statistically significant variations between any specific pairs of groups.

### 3.2. Analysis of the Genetic Variability of T. magnatum Picco Populations

The single-locus marker SCAR A21-inf was analyzed to identify single nucleotide polymorphisms (SNPs) and assess (at least in part) the genetic variability within and among the four white truffle populations from Molise region. In particular, the detection of two SNPs in this polymorphic region [22] allowed to define three distinct haplotypes (I, II, and III).

The populations of *T. magnatum* Picco from the municipalities of Castel del Giudice and Pietrabbondante were characterized by the presence of only two haplotypes, I and II (Table 1). In contrast, truffles from Agnone and Carovilli exhibited all three haplotypes. Fisher’s exact test (performed to assess the association between provenance and haplotype) indicated no significant association between these variables.

### 3.3. Profiling of Prokaryotic and Fungal Communities Associated with T. magnatum Picco Ascocarps Through Next-Generation Sequencing

Next-Generation Sequencing enabled a detailed characterization of the microbial communities associated with the inner tissues of *T. magnatum* Picco fruiting bodies collected from the four natural truffle grounds in the Molise region. A total of 1,243,621 reads for the V3-V4 regions of 16S rDNA and 4,414,966 reads for ITS2 region, resulting in the identification of 622 and 125 Amplicon Sequence Variants (ASVs), respectively, were obtained. The mean number of reads and ASV per sample were 62,181.1 and 68.9 for the V3-V4 regions of 16S rDNA, and 220,748.3 and 15.85 for ITS2 region.

**Figure 3:**
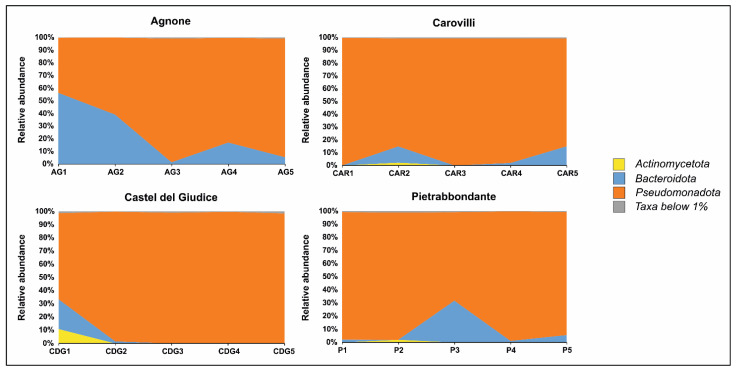
Bacterial community composition of *T. magnatum* Picco ascomata at phylum level.

No archaeal sequences were detected in any of the ascocarps analyzed. On the other hand, bacterial community profiling at the phylum level (Figure 3) revealed a dominance of *Pseudomonadota* and *Bacteroidota* in samples from Agnone, together accounting for an average of 99.59% of the total bacterial community. *Pseudomonadota* was the predominant phylum in samples AG2, AG3, AG4, and AG5, whereas *Bacteroidota* were most abundant in sample AG1.

In truffles collected from the municipality of Carovilli, *Pseudomonadota* was the most represented phylum, with relative abundance values ranging from 84.49% (sample CAR5) to 99.70% (sample CAR1). *Bacteroidota* were present in samples CAR2, CAR4, and CAR5, with relative abundances of 12.71%, 1.88%, and 14.89%, respectively.

The fruiting bodies of *T. magnatum* Picco from Castel del Giudice showed a similar pattern, with *Pseudomonadota* comprising more than 98% of the bacterial community in samples CDG2, CDG3, CDG4, and CDG5. In sample CDG1, although *Pseudomonadota* remained the most abundant phylum (65.22%), *Bacteroidota* and *Actinomycetota* were also prominent, accounting for 22.51% and 10.84% of the community, respectively.

In ascocarps collected from Pietrabbondante, *Pseudomonadota* and *Bacteroidota* were the two most abundant phyla in samples P1, P3, P4, and P5, together comprising an average of 99.37% of the bacterial community. In sample P2, *Pseudomonadota* remained dominant (96.96%), followed by *Actinomycetota* (1.95%).

**Figure 4:**
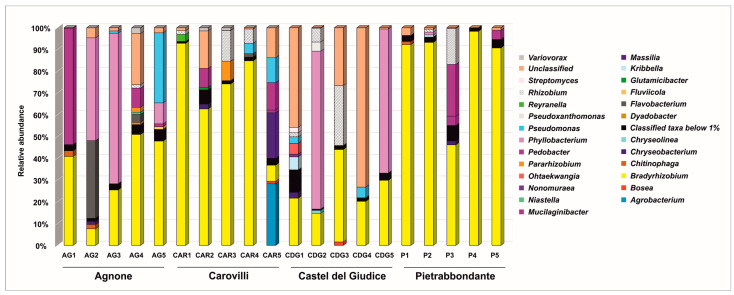
Bacterial community composition of *T. magnatum* Picco ascomata at genus level.

At the genus level (Figure 4), *Bradyrhizobium* was the most abundant classified taxon in all samples from Pietrabbondante, with relative abundances ranging from 46.19% to 98.37%. This genus also predominated in four samples from Carovilli (CAR1, CAR2, CAR3, and CAR4), where its relative abundance varied from 62.69% to 92.87%. In contrast, CAR5 bacterial community was dominated by *Agrobacterium* (28.27%), *Massilia* (20.91%), *Pedobacter* (12.59%), and *Pseudomonas* (11.43%), with *Bradyrhizobium* ranking fifth (7.37%).

Two ascocarps from Castel del Giudice (CDG1 and CDG4) exhibited a high proportion of sequences unclassified at the genus level (45.79% and 73.22%, respectively), highlighting the need for further investigation to fully resolve the bacterial diversity within *T. magnatum* fruiting bodies. Nevertheless, *Bradyrhizobium* was still a prominent genus in this population, with relative abundances ranging from 14.71% (CDG2) to 42.49% (CDG3). In samples CDG2 and CDG5, *Phyllobacterium* was the dominant genus, accounting for 72.57% and 66.20% of the community, respectively.

*Bradyrhizobium* and *Phyllobacterium* were among the dominant genera also within *T. magnatum* Picco ascomata from Agnone site. Some of these samples also showed high percentages of bacteria belonging to *Pedobacter* (53.53% in AG1), *Flavobacterium* (35.69% in AG2), and *Pseudomonas* (32.20% in AG5) genera.

**Figure 5:**
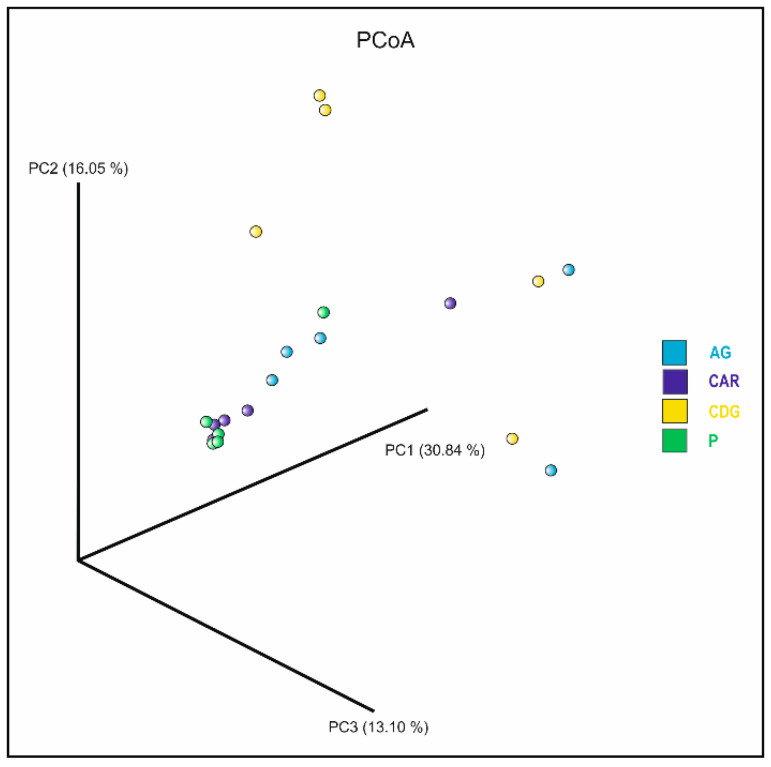
Plot of the Principal Coordinate Analysis (PCoA) based on Bray–Curtis metric, showing the bacterial communities associated with ascocarps collected from Agnone (AG; light blue), Carovilli (CAR; blue), Castel del Giudice (CDG; yellow), and Pietrabbondante (P; green).

Alpha diversity, as measured by the Shannon index, yielded average values of 2.28, 1.95, 1.97, and 1.15 for samples collected from Agnone, Carovilli, Castel del Giudice, and Pietrabbondante, respectively. However, the Kruskal–Wallis test indicated no statistically significant differences in microbial alpha diversity among the examined populations. Beta diversity was assessed using the Bray–Curtis metric to compare the bacterial communities associated with the 20 ascocarps collected from the four study sites. Principal Coordinate Analysis (PCoA) revealed a closer clustering of samples from Pietrabbondante and Carovilli, despite the presence of some outliers (Figure 5). In contrast, samples from Agnone and Castel del Giudice showed greater dispersion. Although the global PERMANOVA indicated a statistically significant difference in the community structure among groups (*p* < 0.05), subsequent pairwise comparisons did not yield any significant results after correcting for multiple testing. Indeed, the resulting q-values were all greater than 0.05. This result implies that, although differences exist at the group level overall, the individual pairwise comparisons did not allow us to detect the groups that differed significantly from each other.

Nevertheless, differential abundance analysis conducted using the Analysis of Compositions of Microbiomes with Bias Correction (ANCOM-BC) at the genus level identified various known and unclassified genera that were significantly differentially abundant between *T. magnatum* Picco population pairs (Table S1). It is likely that these taxa contribute to the observed heterogeneity in community composition across samples.

The analyses carried out on the gleba fungal communities, as expected, revealed a predominance of the genus *Tuber* across all samples examined. In several cases—specifically in samples AG2, CAR4, CAR5, CDG4, and P5—*Tuber* was the only fungal genus detected through Next-Generation Sequencing. However, as shown in Table 2, additional fungal genera were identified in the remaining truffle samples, albeit generally at very low relative abundances. More specifically, samples AG1, AG3, AG4, and AG5, all collected from Agnone area, contained fungi belonging to the genera *Exophiala*, *Pseudodictyosporium*, and *Helvella*. The genus *Exophiala* was also identified in ascocarps CAR1 and CAR3, along with the genera *Thyridium*, *Dactylospora*, *Capronia*, and *Penicillium*. In samples from Castel del Giudice (CDG1, CDG3, CDG5), in addition to *Tuber*, several other fungal genera were associated with the fruiting bodies, including *Exophiala*, *Pseudodictyosporium*, *Helvella*, *Helvellosebacina*, *Tomentella*, *Anthopsis*, *Thyridium*, *Pseudocosmospora*, and *Penicillium*. Truffles collected from Pietrabbondante (P1, P2, P3, and P4) revealed the presence of *Exophiala*, *Dactylonectria*, *Dactylospora*, *Capronia*, *Minimelanolocus*, *Tomentella*, *Anthopsis*, *Thyridium*, and *Sebacina*. However, also for fungi, sequences unclassified at genus level were found in some samples (Table 2).

**Figure 6:**
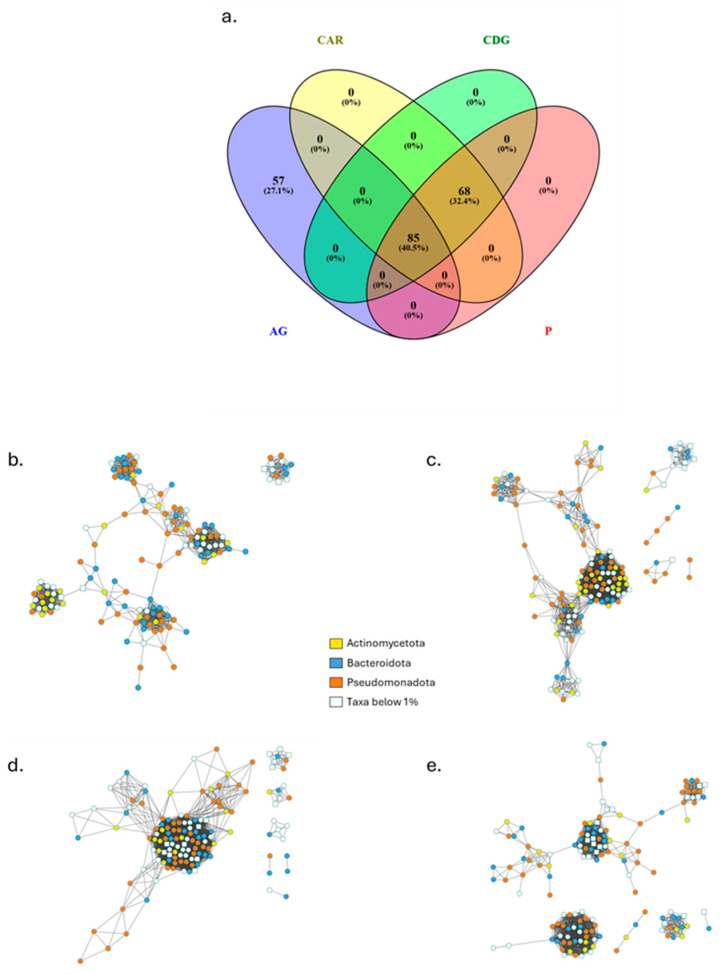
Overlap among taxa of the individual truffle populations (**a**) and the related networks of Agnone (AG; (**b**)), Carovilli (CAR; (**c**)), Castel del Giudice (CDG; (**d**)) and Pietrabbondante (P; (**e**)) samples. Round and square nodes represent bacterial and fungal species, respectively. Colors trace the main abundant phyla.

**Figure 7:**
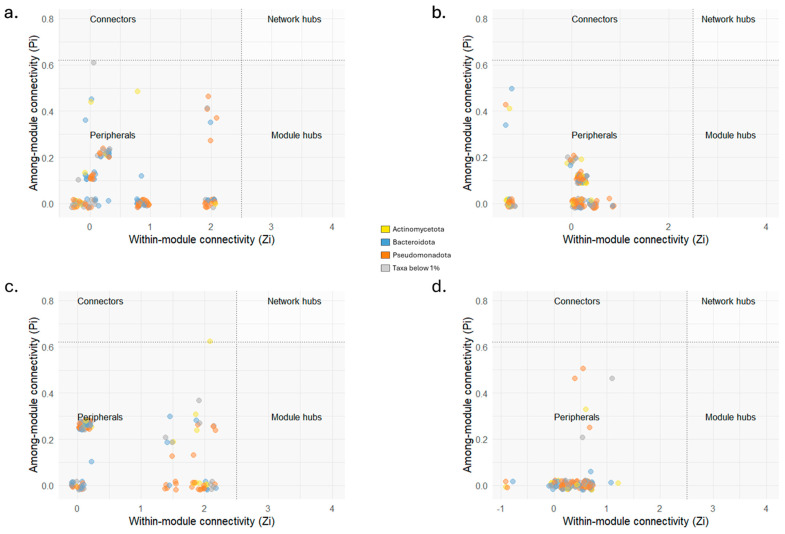
Zi-Pi scatterplots showing the classification of nodes to identify potential keystone taxa in the four truffle populations from Agnone (AG; (**a**)), Carovilli (CAR; (**b**)), Castel del Giudice (CDG; (**c**)), and Pietrabbondante (P; (**d**)) in peripherals, modular hubs, connectors or network hubs. Module connectors are above the horizontal dashed line (Pi = 0.62), while module hubs are at the right of the vertical dashed line (Zi = 2.5). Network hubs are in the top right area. Dot colors represent the phyla to which the different taxa belong.

Network-based approach, used to model complicated community assemblies, showed distinct networks of bacterial and fungal OTUs from the truffle individual populations (Figure 6) and all measured parameters were higher compared to random networks (Table 3). This validates the characteristics of the networks reconstructed on sequencing data and establishes them as good non-random models to investigate the related bacterial and fungal communities. A small subset of species within the community can be identified as their presence and activity are able of shaping the community structure through strong interactions with the environment or with other taxa. Their removal has effects on the whole community assembly resulting in potential devasting of its maintenance and functioning. The number of these taxa was equal to 142 in AG, 153 in CAR, 140 in CDG, and 147 in P. The overlap highlighted 57 taxa exclusively present in AG samples (Figure 6a). The fungal taxa were represented in numbers equal to 6, 8, 14, and 18, respectively for AG, CAR, CDG, and P (Figure 6b–e). Not only the numbers and overlap among bacterial and fungal taxa, but also the network characteristics were evaluated (Table 3). The average path length (APL) was higher in AG (5.04), in P was equal to 3.87, in CAR equal to 3.09, and 1.96 in CDG. Also, the modularity showed the higher value in AG (0.78), followed by P (0.66), CAR (0.43), and CDG (0.12). The average clustering coefficient (ACC), instead, showed the higher value in CDG (0.91), followed by CAR, P (0.88 for both), and AG (0.85). The modularity observed was associated with 7, 8, 10, and 8 modules identified for AG, CAR, CDG, and P networks, respectively, with no hubs being detected (Figure 7). Additionally, the taxa were mainly classified as peripheral taxa (Table 3, Figure 7). Only in the CDG network, a taxon belonging to the phylum *Actinomycetota* was classified as connector among modules (Figure 7c) and can be considered a keystone taxon of the community.

## 4. Discussion

*Tuber magnatum* Picco is globally recognized as a premium truffle species, prized for its exceptionally high market value, which can reach several thousand euros per kilogram depending on the harvest year and fruiting body size [39]. Given the risk of food fraud associated with this highly sought-after fungus, detailed analysis and characterization of this species are crucial.

The Molise region, located in Central-Southern Italy, is a key contributor to truffle production, accounting for approximately 40% of the national yield of both black and white truffles [4]. Interestingly, “Alto Molise” woodlands are particularly rich in *T. magnatum* fruiting bodies [4]. Nevertheless, this region remains underrepresented in scientific literature compared to other territories. The sustainable valorization of Molise truffle resources could offer a strategic opportunity to promote food-related tourism, increase visitor inflow, and support the economic development of a region that faces considerable socio-economic challenges [40].

In view of these considerations, the present study sought to expand the current understanding of Molise white truffle by examining the morphological, genetic, and microbiological characteristics of *T. magnatum* Picco populations collected from four distinct areas within the region.

Differently from previous findings by Monaco et al. [4,20], which reported significant morphological differences between white truffles collected from two study areas in Molise region, this research found no statistically significant differences for weight and maturation stage. These discrepancies likely reflect differences in sampling timeframes: Monaco et al. [4,20] collected specimens at different stages of the harvesting season (November 2019 and January 2020), whereas all samples in this study were collected within the same temporal window (late November–early December).

For the peridium thickness, although the Kruskal–Wallis test indicated an overall significant variation among truffle groups, the subsequent pairwise comparisons with Dunn’s test and Bonferroni correction did not identify any specific population pairs with significant differences. Nevertheless, unadjusted (raw) *p*-values suggested potential distinctions between truffles from Castel del Giudice and Agnone and between Agnone and Pietrabbondante, but these variations were not sustained after adjustments. Accordingly, additional studies are necessary to investigate the environmental, genetic, or ecological factors driving the observed patterns.

Interestingly, genetic analysis focused on the single-locus marker SCAR A21-inf revealed the presence of the haplotype III only in some *T. magnatum* samples from both Agnone and Carovilli municipalities. This result corroborates earlier findings reported by Mello et al. [22] and Monaco et al. [4], who detected this haplotype exclusively in Molise white truffle samples. Haplotypes I and II were found in all four populations. Fisher’s exact test showed no significant association between haplotype distribution and sampling sites, suggesting that haplotype III (as well as haplotypes I and II) is not limited to specific collection areas, but may be distributed across multiple populations within Molise. Although further studies involving larger sample sizes and broader geographic coverage are needed to clarify the distribution patterns and evolutionary significance of this haplotype within *T. magnatum* populations, the current findings support the hypothesis that it may represent to date a region-distinctive genetic feature.

Understanding the genetic profile of truffle populations is critical not only for advancing scientific knowledge, but also for ecological considerations, conservation efforts, and the traceability of the commercially valuable species [14,16]. Similarly, characterizing the truffle-associated microbiota holds significant promise for identifying specific bacterial taxa that could serve as reliable biomarkers for tracing the geographical origin of truffles [41,42]. In this regard, the present research contributed to broadening the current information on truffle microbiology, by analyzing the microbial communities (both prokaryotic and fungal) associated with 20 *T. magnatum* ascomata.

It is well established that diverse bacterial taxa inhabit the ascoma of *T. magnatum* [4,41,43,44,45,46], as well as other *Tuber* species [23,47]. Most belong to the phylum *Pseudomonadota*, particularly the classes *Gammaproteobacteria* and *Alphaproteobacteria* [46]. Consistent with previous studies [12,48,49,50], *Bradyrhizobium*, a genus within the *Alphaproteobacteria*, was identified as the most abundant classified bacterial genus in the majority of truffle ascomata here analyzed. Barbieri et al. [44] hypothesized a potential role for *Bradyrhizobium* in nitrogen nutrition of *T. magnatum*, detecting the presence of the nitrogenase gene *nifH* from *Bradyrhizobium* spp. within truffle ascomata and reporting nitrogen fixation activity comparable to that observed in early-stage legume nodules colonized by symbiotic nitrogen-fixing bacteria [44,51]. Recently, Graziosi et al. [46] described beneficial interactions between *T. magnatum* mycelium and *Bradyrhizobium* spp., which allow the in vitro growth of *T. magnatum* mycelium.

Interestingly, the bacterial communities of some of the examined samples were dominated by the genera *Pedobacter*, *Phyllobacterium*, and *Agrobacterium*, rather than *Bradyrhizobium*.

*Pedobacter* species are widely distributed across diverse habitats, including terrestrial [52] and marine environments [53], and in association with higher organisms [54]. Notably, *Pedobacter* has been reported as one of the main bacterial genera found within the fruiting bodies of both *T. aestivum* Vittad. [55] and *T. magnatum* Picco [12]. Members of this genus are notable for their potential to produce industrially relevant enzymes [56,57] and antibiotics with potent activity against multidrug-resistant pathogens, such as the cyclic lipodepsipeptides pedopeptins and isopedopeptins [58]. Moreover, the ability of some *Pedobacter* species to produce chitinases that degrade the fungal cell walls suggests their potential involvement in truffle ascocarp degradation and spore dispersal [55]. *Phyllobacterium* spp. are commonly associated with plants but are also found in various environments such as water, soil, rhizosphere, root nodules, and even in association with single-celled organisms [59,60,61,62,63,64]. The presence of *Phyllobacterium* has also been documented in *T. aestivum*, where species belonging to this bacterial genus have been isolated from ectomycorrhizae [65]. Regarding *Agrobacterium*, this genus was historically defined by plant pathogenicity as a key phenotypic trait; however, this perspective has shifted to encompass non-pathogenic species too, following a redefinition of the genus grounded in molecular taxonomy [66,67].

Alpha diversity metrics indicated no significant differences in microbial richness and evenness among the four examined *T. magnatum* populations. In contrast, beta diversity analyses revealed significant variation in bacterial community composition across sites, although pairwise comparisons did not detect statistically significant differences, likely due to the high variability within truffle populations. Therefore, while certain bacterial taxa are shared across Molise truffles, localized environmental factors and microhabitat conditions likely play a key role in shaping the fruiting body-associated microbiota [42] and contribute to the observed heterogeneity, as further supported by the differential abundance analysis. Moreover, in some samples, both at the genus and species levels, many unclassified taxa were detected, indicating that further efforts are needed to unravel the still largely unexplored bacterial diversity within *T. magnatum* ascocarps, underscoring the need for further research.

To date, most studies describing the microbial communities associated with truffle ascomata have focused on bacteria, whereas fungi have been scarcely investigated. This is chiefly attributable to the difficulties related to the massive presence of *Tuber* DNA, which can interfere and hinder the amplification, sequencing, and detection of “exogenous fungi” within the gleba [41,68]. Therefore, as expected, NGS-based fungal community profiling showed a pronounced dominance of *T. magnatum* DNA within the 20 ascocarp samples, which substantially constrained the detection of co-occurring fungal taxa. Despite this limitation, various fungal genera were identified, providing preliminary insights into the mycobiota associated with *T. magnatum* fruiting bodies. Among fungi, *Exophiala* was detected across all Molise sampling sites, consistent with the observations of Marozzi et al. [68], who also documented the presence of *Dactylonectria*, *Minimelanolocus*, and *Tomentella* in ascocarps from other Central Italian regions. *Tomentella* is an important component of ectomycorrhizal communities and it was widely found in a *T. magnatum* truffle-ground by molecular typing of the below-ground fungal community [69]. Additionally, the fruiting bodies collected from Molise region contained further fungal taxa, among which the genus *Sebacina*, known to contain ectomycorrhizal species. Considering these findings, ecological functions and interactions of the mycobiota within the ascocarp microenvironment warrant detailed characterization to elucidate its potential roles in truffle biology and ecosystem dynamics.

Also, the analysis of the properties of the network derived for the individual populations confirmed a variability among truffle populations and a role of the fungal taxa in truffle biology and ecosystem dynamics, supported by the comparison with randomized data. Majority taxa of the truffle populations were classified as peripherals, which represent specialists in the community [37]. These type of community members play a key role to respond to niche differentiation and modulate functional traits [70], thus suggesting a specialization of the communities that is site-dependent.

As known, bacterial and fungal taxa in truffles are associated with aroma formation, ascocarp maturation, and nutrient fixation and are recruited from the soil under the hypotheses of an effect mediated by the host tree [18]. Thus, future studies may benefit from combining the morphological, genetic, and microbiological characteristics of ascocarps with host plants and phytosociological surveys of the sampling sites, also including the relatively low-abundant species that may play pivotal roles within the communities, as shown by our results.

## 5. Conclusions

Our findings showed that *T. magnatum* populations from Molise seem to exhibit some consistent traits across the region. The presence of a unique genetic feature found to date only in Molise truffles opens new questions on its origin that could be associated with not yet observed physical traits or to specific abilities to be investigated. Complex and heterogeneous microbial communities, including many unclassified taxa, indicates the need for extensive further investigations. In fact, although no statistically significant differences were identified between any of the examined group pair, the pronounced heterogeneity supports the possibility that, with additional research, markers of provenance could be identified to discriminate *T. magnatum* populations at both the regional and local scale. Regional markers might be derived by exploiting traits that are consistently shared among populations from the region, whereas local markers could be identified by leveraging the heterogeneity observed among the populations investigated.

In conclusion, this research highlights the potential to identify multi-level traceability markers for *T. magnatum* at regional and local scale. As Molise region is one of the major contributors to the national production of the prized white truffle, advancing the understanding of local truffle populations from a scientific point of view is essential. Indeed, such efforts will support sustainable management practices, stimulate regional economic growth, and safeguard the authenticity of this high-value species against food fraud. Integrating genetic, microbiological, and metabolic research will be critical to fully unlock the ecological and economic potential of Molise white truffles.

## Figures and Tables

**Table 1:** Main morphological and genetic features of the investigated *T. magnatum* Picco fruiting bodies. ^1^ Kruskal–Wallis test for fruiting body weight: *p* > 0.05. ^2^ Kruskal–Wallis test for peridium thickness: *p* < 0.05. Post Hoc pairwise comparisons conducted using Dunn’s test with Bonferroni correction: *p* > 0.05. ^3^ Kruskal–Wallis test for maturity stage: *p* > 0.05. ^4^ Fisher’s exact test for association between haplotype and provenance: *p* > 0.05. * Values in brackets indicate the percentage of asci containing mature spores (mean values).

Collection Area	Sample Code	Weight (g) ^1^	Peridum Thickness ^2^ (µm)	Maturity Stage ^3,^*	Scar A21-inf Haplotype ^4^
Agnone (AG)	AG1	6.20	264.71	III (95%)	II
	AG2	5.40	301.95	II (45%)	I
	AG3	3.34	308.48	I (2%)	I
	AG4	3.35	325.18	I (0%)	III
	AG5	4.73	394.80	I (4%)	II
Carovilli (CAR)	CAR1	5.22	240.69	III (95%)	II
	CAR2	4.20	234.63	I (0%)	I
	CAR3	5.28	260.32	III (93%)	III
	CAR4	4.44	344.98	II (25%)	I
	CAR5	4.95	227.31	I (1%)	III
Castel del Giudice (CDG)	CDG1	7.73	198.12	III (99%)	I
	CDG2	6.51	209.99	III (96%)	I
	CDG3	7.83	311.39	II (50%)	II
	CDG4	4.09	222.33	II (59%)	I
	CDG5	3.69	229.27	II (20%)	I
Pietrabbondante (P)	P1	3.25	268.03	III (90%)	I
	P2	2.75	224.38	III (85%)	II
	P3	6.19	182.80	III (87%)	I
	P4	5.64	236.44	III (86%)	II
	P5	7.84	235.03	III (90%)	I

**Table 2:** Main fungal genera found in the investigated *T. magnatum* Picco fruiting bodies.

	Agnone					Carovilli					Castel del Giudice					Pietrabbondante				
Fungal Genera	AG1	AG2	AG3	AG4	AG5	CAR1	CAR2	CAR3	CAR4	CAR5	CDG1	CDG2	CDG3	CDG4	CDG5	P1	P2	P3	P4	P5
*Anthopsis*															+			+		
*Capronia*								+										+		
*Dactylonectria*																+			+	
*Dactylospora*								+									+	+		
*Exophiala*	+					+		+			+					+	+	+		
*Helvella*			+	+									+							
*Helvellosebacina*											+									
*Minimelanolocus*																		+		
*Penicillium*								+			+									
*Pseudocosmospora*											+									
*Pseudodictyosporium*			+		+										+					
*Sebacina*																	+			
*Thyridium*						+		+							+		+	+		
*Tomentella*															+			+		
*Unclassified*	+		+	+			+	+			+	+				+	+	+	+	

**Table 3:** Empirical networks comparison with random networks.

	Empirical Networks							Random Networks (Avg ± SD)		
	taxa	Edges	Mean Degree	Density	APL *	Modularity	ACC **	APL *	Modularity	ACC **
**AG**	142	1082	15.24	0.1080	5.04	0.78	0.85	2.14 ± 0.0083	0.21 ± 0.0055	0.19 ± 0.010
**CAR**	153	2043	26.71	0.1757	3.09	0.43	0.88	1.87 ± 0.0025	0.14 ± 0.0048	0.28 ± 0.009
**CDG**	140	3138	44.83	0.3224	1.96	0.12	0.91	1.71 ± 0.0001	0.09 ± 0.0035	0.41 ± 0.007
**P**	147	1469	19.99	0.1369	3.87	0.66	0.88	2.01 ± 0.0068	0.18 ± 0.0054	0.22 ± 0.010

* average path length; ** average clustering coefficient.

## Data Availability

SCAR A21-inf sequences have been deposited in the GenBank database (accession numbers from PV754620 to PV754639). 16S rDNA and ITS sequences generated in the present study have been deposited in the NCBI Sequence Read Archive under the accession number PRJNA1272064. All other data generated under this work, if not present in the manuscript, are available under request to the corresponding author (gabriella.sferra@unimol.it).

## Supplementary Materials

The following supporting information can be downloaded at: https://www.mdpi.com/article/10.3390/microorganisms13102340/s1, Table S1: Differentially abundant classified genera between population pairs of *T. magnatum* Picco from “Alto Molise”.
