# Supplementary material for: Morphological, Genetic, and Microbiological Characterization of Tuber magnatum Picco Populations from “Alto Molise”, Central-Southern Italy
Source: Microorganisms. 2025 Oct 11;13(10):2340. doi: 10.3390/microorganisms13102340 (PMC12565911; doi:10.3390/microorganisms13102340)
Supplement: Supplementary file 1 [file microorganisms-13-02340-s001.zip › microorganisms-3897188-supplementary.pdf]

Table S1. Differential abundance analysis at the genus level assessed using the Analysis of Compositions of Microbiomes with Bias Correction (ANCOM-BC) method.

Differentially abundant genera	AG - CAR	AG - CDG	AG - P	CDG - CAR	CDG - P	P - CAR
Acidibacter	-	D - E	-	-	-	-
Acinetobacter	-	D - E	-	-	-	-
Actinophytocola	-	D -E	-	E - D	-	-
Bradyrhizobium	-	-	D - E	-	D - E	-
Candidatus_Udaeobacter	-	D - E	-	-	-	-
Caulobacter	-	D - E	-	-	-	-
Chthoniobacter	-	D - E	-	E - D	-	-
Dactylosporangium	-	D - E	D - E	E - D	-	E - D
Dongia	-	-	-	-	-	E - D
Edaphobacter	-	D - E	-	-	-	-
Ellin6067	-	-	-	E - D	-	-
Flavobacterium	-	E - D	E - D	-	-	-
Fluviicola	-	-	-	E - D	-	-
Glutamicibacter	-	D - E	-	-	-	-
Gynurincola	-	-	D - E	-	-	-
Inquilinus	-	-	-	-	-	E - D
Jiangella	-	-	-	E - D	-	-
Kribbella	E - D	D - E	-	E - D	-	E - D
Lentzea	-	D - E	D - E	-	-	-
Luteolibacter	-	E - D	-	-	-	-
Lysobacter	-	D - E	-	-	-	-
Methylobacterium	-	D - E	-	E - D	-	-
Paenisporosarcina	-	D - E	-	E - D	-	-
Pajaroellobacter	-	-	D - E	-	-	-
Paludibaculum	-	-	-	E - D	-	-
Pedobacter	-	E - D	-	-	-	-
Promicromonospora	-	-	D - E	-	-	-
Pseudomethylobacillus	-	-	-	E - D	-	-
Pseudonocardia	-	D - E	-	E - D	-	-
Pseudorhodoplanes	-	D - E	-	E - D	-	-
Rhizobacter	-	D - E	-	-	-	-
Rhizobium	-	D - E	-	-	-	-
Rhodomicrobium	-	D - E	-	E - D	-	-
Roseateles	-	-	-	E - D	-	-
Staphylococcus	-	D - E	-	-	-	-
Streptomyces	-	D - E	-	-	-	-
Tahibacter	-	-	-	E - D	-	-
TM7a	-	D - E	D - E	-	-	-
Variovorax	-	E - D	-	-	-	-

In the table only the differentially abundant classified genera are reported. E: enriched. D: depleted
